# Predictive Value of Quantitative ADC, SUVmax, and the SUVmax/ADC Ratio for Biological Behavior and Prognosis in High-Risk Prostate Cancer

**DOI:** 10.3390/jcm14207150

**Published:** 2025-10-10

**Authors:** Abdullah Enes Ataş, Ülkü Kerimoğlu, Zeki İlhan, Şeyma Ünüvar, Özlem Şahin, Zeynep Aydın, Hacı Hasan Esen

**Affiliations:** 1 Department of Radiology, Necmettin Erbakan University, Konya 42090, Türkiye; kerimogluulku@yahoo.com; 2 Department of Radiology, Sana Klinikum Hof, 95032 Hof, Germany; dr.zeki.ilhan@gmail.com; 3 Department of Radiology, Konya City Hospital, Konya 42020, Türkiye; seymababaoglu@hotmail.com; 4 Department of Nuclear Medicine, Necmettin Erbakan University, Konya 42090, Türkiye; drozlemsahin@gmail.com; 5 Department of Nuclear Medicine, Konya City Hospital, Konya 42020, Türkiye; drzaydin42@hotmail.com; 6 Department of Pathology, Necmettin Erbakan University, Konya 42090, Türkiye; drhasanesen@gmail.com

**Keywords:** prostate cancer, magnetic resonance imaging, positron emission tomography

## Abstract

**Background/Objectives**: To investigate the importance of ADC, SUVmax, and SUVmax/ADC values in the prognosis and biological behavior of prostate cancer. **Methods**: In this retrospective study, ADC measurements in diffusion MRI were made by two radiologists by correlating the lesions with the highest SUVmax value from Ga-68 PSMA PET/CT examinations of 81 patients with prostate cancer. The quantitative values were compared with histopathological grade, presence of perineural invasion, and lymph node and bone metastasis. **Results**: For D’Amico high-risk patients, a statistically significant difference among the ADC, SUVmax, and SUVmax/ADC measurements was reported (*p* < 0.001). Cut-off values were defined as 0.52 (×10^−3^ mm^2^/s) for ADC, 9.73 for SUVmax, and 20.28 for the SUVmax/ADC ratio (AUC = 0.887, 0.747, 0.817, respectively) for the high-risk categories. The Youden indices were 0.643, 0.405, and 0.437, respectively. In logistic regression, the SUVmax/ADC ratio was a significant predictor of the high-risk group (AUC = 0.844, *p* = 0.002), demonstrating superior performance to a model with individual ADC and SUVmax values (AUC = 0.796, *p* = 0.006). For the advanced-grade group, the SUVmax and SUVmax/ADC ratios differed significantly (*p* < 0.001). The CAPRA score showed significant correlations with all imaging biomarkers: negatively with ADC (rho = −0.456, *p* < 0.001) and positively with SUVmax (rho = 0.359, *p* = 0.001) and the SUVmax/ADC ratio (rho = 0.441, *p* < 0.001). The presence of perineural invasion had no significant correlation with any of the variables (*p* > 0.05). The presence of bone metastases and PSA and free PSA levels differed significantly (*p* = 0.003, *p* = 0.001, respectively). In the presence of lymph node metastasis, SUVmax and SUVmax/ADC ratios were found to be significant (*p* = 0.019, *p* = 0.01, respectively). In the survival (OS) analysis, a low ADC value was found to be associated with shorter survival (median OS: 61 vs. 106 months). **Conclusions**: Among advanced-grade and high-risk prostate cancer patients, ADC, SUVmax, and SUVmax/ADC values can be employed as alternative prognostic factors for predicting the biological behavior of the disease.

## 1. Introduction

Local invasion and distant metastasis are the factors affecting prognosis and survival in prostate cancer. The parameter that determines the local staging is the extraprostatic spread of the tumor focus. The histopathological equivalent of this is the presence of perineural invasion [1]. Bone metastasis is common throughout the course of the disease; it affects morbidity and mortality [2].

As noninvasive imaging methods have become widespread in the imaging of prostate cancer, it is necessary to use some factors to predict the behavior of the disease. PSA (prostate-specific antigen) value and Gleason ISUP (International Society of Urogenital Pathologists)-grade groups are used in determining risk groups in clinical practice [3,4,5].

Accurate risk stratification is the cornerstone of modern prostate cancer management, yet it remains a significant clinical challenge. While risk groups are traditionally defined by serum PSA levels, clinical stage, and ISUP Grade Group, the most recent European Association of Urology (EAU) guidelines underscore the increasing complexity and heterogeneity within these categories [5]. A critical evolution in this framework is the subdivision of the intermediate-risk group into ‘favorable’ and ‘unfavorable’ subsets, a distinction that acknowledges the vastly different prognoses within what was once a monolithic category [5,6]. This stratification is crucial, as it directly influences the decision between active surveillance and definitive treatment [7]. Furthermore, the distinction between unfavorable intermediate-risk and high-risk disease is pivotal for determining the need for multimodal therapies. However, this stratification still relies heavily on biopsy findings, which are prone to sampling errors and may not fully capture the biological aggressiveness of the entire tumor. This highlights an urgent need for non-invasive biomarkers that can provide a more holistic and accurate assessment of tumor biology, thereby refining risk stratification and guiding more personalized therapeutic strategies [8].

While these tools are foundational in clinical practice, they possess inherent limitations that can lead to suboptimal patient management. For instance, PSA levels can be elevated in benign conditions such as prostatitis or benign prostatic hyperplasia, leading to a high false-positive rate and unnecessary biopsies. Conversely, some aggressive tumors may present with deceptively low PSA levels [9]. More significantly, Gleason score, while a powerful prognostic indicator, is susceptible to sampling errors and inter-observer variability, potentially leading to undergrading of the tumor’s true aggressiveness. This is particularly challenging in heterogeneous tumors where the most aggressive clones may be missed by standard biopsy schemes, thereby misclassifying a patient’s risk and delaying appropriate definitive therapy [10].

Multiparametric prostate magnetic resonance imaging (mpMRI) is an important imaging method used in the detection of prostate cancer, in biopsy guidance, and in posttreatment follow-up. Apart from routine conventional sequences, functional sequences such as diffusion-weighted imaging and dynamic contrast-enhanced imaging are also used to reveal the lesion. In particular, diffusion-weighted images and apparent diffusion coefficient (ADC) maps obtained from them are among the methods that greatly contribute to diagnoses [11,12].

Molecular methods are also becoming increasingly important for primary focus detection, local and systemic staging, bone metastasis detection, and recurrence follow-up of prostate cancer. Among these, gallium-68 prostate-specific membrane antigen positron emission tomography (Ga-68 PSMA PET) applications are imaging methods that have been frequently used in recent years, especially in intermediate- and high-risk cases, and are more sensitive than conventional methods [13,14,15].

The integration of these two powerful modalities offers a synergistic approach: diffusion-weighted imaging (DWI) delineates tissue cellularity and microstructure [16,17]. while PSMA PET/CT reveals its molecular phenotype and metabolic activity. This dual assessment provides a more comprehensive biological profile of the tumor, forming a robust basis for accurate staging and characterization of disease aggressiveness [18,19].

The aim of this study is to determine whether quantitative measurements play a role in prognosis by examining the relationship between SUVmax (standardized uptake volume) value (measured in Ga-68 PSMA PET/CT) with histopathological grade, perineural invasion, and bone metastasis, and ADC value (measured in mpMRI) for staging in prostate cancer cases.

## 2. Materials and Methods

### 2.1. Patient Selection

In this retrospective study, 313 patients who underwent Ga-68 PSMA PET/CT imaging for prostate cancer staging in the tertiary university hospital between September 2018 and December 2020 were evaluated. Of these patients, 232 patients who did not have multiparametric prostate MRI or had no pathology results were excluded from the study. As a result, 81 patients with histopathologically proven prostate cancer who underwent multiparametric prostate MRI and Ga-68 PSMA PET/CT were included in the study.

Six patients with ISUP Grade 1 disease who underwent PSMA PET/CT for staging due to Gleason-discordant elevated PSA levels or suspicion of bone metastases were included in the study.

The first images of the patients were evaluated before treatment. MRI and PET/CT scans, less than two months apart, were evaluated by correlation.

### 2.2. PET/CT Evaluation

In the nuclear medicine clinic, Ga-68-labeled PSMA was synthesized as Glu-NH-CONH-Lys(Ahx)-HBED-CC (DKFZ-PSMA-11 or PSMA-HBED) using disposable cassettes and using a fully automated synthesis system (Modular Lab PharmTracer, Eckert & Ziegler Eurotope GmbH, Berlin, Germany). Ga-68Cl3 required for synthesis was obtained by using 6 mL of 0.1 M HCl. A dose of Ga-68 PSMA-11 was given intravenously as 1.8–2.2 MBq/kg (range: 0.049–0.06 mCi/kg).

Examinations were conducted with a PET/CT scanner system (Biograph 6 TruePoint, Siemens, Erlangen, Germany) in the supine position from the base of the skull to the middle of the thigh an average of 63 ± 23 min after the injection of the radiopharmaceutical.

Subsequently, fusion images and maximum intensity projection (MIP) images were obtained with the workstation software Syngo.via VB30A (Siemens, Erlangen, Germany), and the patients were evaluated. Two researchers, one with 15 years of experience and the other with 4 years, examined the images using a workstation to determine SUVmax values for malignant tumor foci in the prostate gland. The lesion with the highest SUVmax value was then accepted as the index/dominant lesion. Additionally, the existence of lymph nodes and bone metastases was assessed.

Patients with bone metastases were stratified into two subgroups based on the number of lesions: those with up to three metastases were classified as ‘oligometastatic’, and those with more than three were classified as ‘polymetastatic’.

### 2.3. MRI Acquisition Protocol

Multiparametric prostate MRI scans using a 1.5 T MRI device (MAGNETOM Aera, Siemens, Erlangen, Germany) were obtained using phased-array pelvic coils (48 channels). To avoid causing artifacts in diffusion-weighted sequences, an enema was applied before the examination. During the protocol, no antiperistaltic agent was used.

Diffusion-weighted images and ADC maps were obtained. For diffusion-weighted images, b values were 50, 400, and 800 s/mm^2^; calculated high b values were 1400 s/mm^2^; TR = 3500 ms; TE = 60 ms; FOV = 18 cm; voxel size was 1.5 × 1.5 × 3.2 mm; and slice thickness was 3 mm.

### 2.4. Radiologic Evaluation

**Figure 1:**
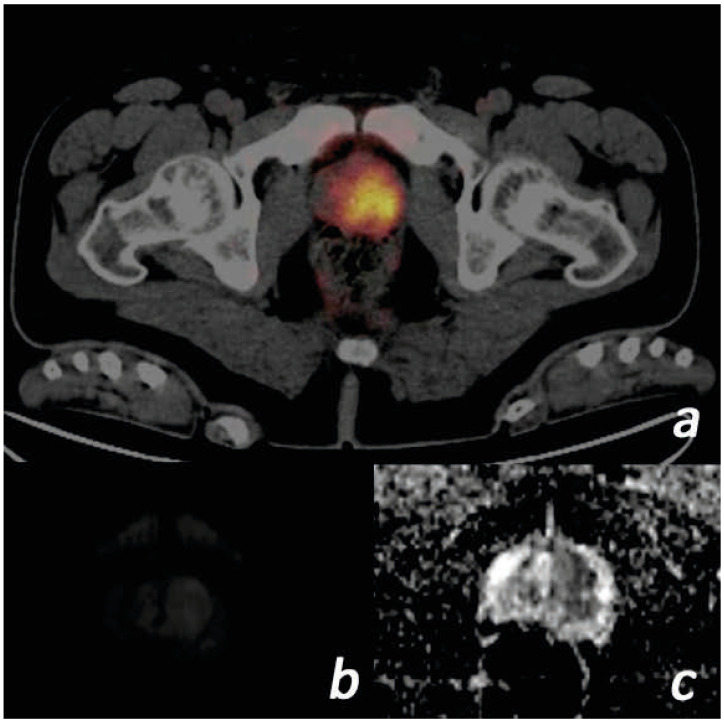
Images of a 57-year-old male patient. (PSA = 117.1 ng/mL, free PSA = 36.2 ng/mL, Gleason score = 4 + 3, ISUP grade = 3) (**a**) Tumor with SUVmax value of 16.38 on the left in the prostate gland transition zone on Ga-68 PSMA PET/CT focus is shown. (**b**) The lesion is slightly hyperintense on diffusion-weighted b = 800 images. (**c**) ADC images are markedly hypointense. The ADC value was measured as 0.40. ADC, apparent diffusion coefficient; ISUP, International Society of Urogenital Pathologists; PET/CT, positron emission tomography/computed tomography; PSA, prostate-specific antigen; PSMA, prostate-specific membrane antigen; SUVmax, standardized uptake value.

After correlation of the index/dominant lesion with Ga-68 PSMA PET/CT fusion images (Figure 1), diffusion-weighted images and ADC maps were evaluated. The mean ADC measurement (×10^−3^ mm^2^/s) of the index/dominant lesion was calculated from ADC maps by two radiologists, one of whom had 20 years and the other 4 years of experience in abdominal radiology. The region of interest (ROI) determined for the measurement was 25 mm^2^ for standard purposes. Due to the possibility that ADC measurements were subjective, intraobserver and interobserver agreement were investigated. The means of all measured ADC values were used in the statistical analysis.

### 2.5. Histopathological Evaluation

Histopathological preparations were obtained by transrectal prostate needle biopsy, transurethral resection, or radical prostatectomy. In patients who underwent transrectal prostate biopsy, tissue sampling from suspicious lesions was performed using MRI-guided cognitive fusion in addition to systematic biopsy. The obtained preparations were evaluated by a pathologist with 15 years of experience in prostate pathology, in accordance with the Gleason scoring and ISUP classification [20]. The histopathological grade of the index lesion was determined, and the presence and absence of perineural invasion criteria were also evaluated.

### 2.6. Statistical Analysis

Intraobserver and interobserver agreement in ADC measurements was investigated using the intraclass correlation coefficient (ICC). For the normality test, histogram analysis and the Shapiro–Wilk test were used. Pearson and Spearman tests were used for the correlation analysis. ANOVA (analysis of variance) was used for comparison with each grade. The t-test was used for data with normal distribution for comparison analysis according to grade, invasion, and bone metastasis, and the Mann–Whitney U test was utilized for data without normal distribution. Logistic regression analysis was performed to predict the high-risk group using quantitative imaging parameters. When a statistically significant difference was detected in the measured values, a receiver operating characteristic (ROC) curve was used to determine the cut-off value, sensitivity, specificity, and positive and negative predictive values. Kaplan–Meier survival curves were used for the survival analysis, and Cox regression analysis was subsequently applied to determine the hazard ratio for parameters that were found to be significant. All statistical data analyses were obtained using the R studio application v3.6.3 and IBM SPSS version 25. *p* < 0.05 was considered statistically significant.

### 2.7. Ethics Committee Approval

Ethics committee approval was obtained for this retrospective study, according to the decision of the Necmettin Erbakan University Non-Pharmaceutical and Non-Medical Device Research Ethics Committee, meeting dated 19 February 2021 and numbered 2021/3109.

## 3. Results

### 3.1. Descriptive Findings

A total of 81 male patients aged 50–86 years (mean 67.54 ± 7.16) were included in the study. The Gleason ISUP grades of the patients were 7.41% (*n* = 6) grade 1, 14.81% (*n* = 12) grade 2, 14.81% (*n* = 12) grade 3, and 13.58% (*n* = 11) grade 4. A total of 49.38% (*n* = 40) of the patients had grade 5 prostate cancer. While 51 of the patients (62.9%) had perineural invasion, bone metastases were detected in 31 patients (38.2%) (Table 1).

ADC values were normally distributed, but all other parameters were not normally distributed.

The lowest ADC measurement made from the patients was 0.22, the highest was 0.77, and the mean value was calculated as 0.49 ± 0.11 mm^2^/s.

The median SUVmax value obtained was 11.47 (4.8–89.76). The median value for the SUVmax/ADC ratio was 24.08 (7.15–280.5). The median value for prostate volume measured from the patients was 53 (16–150) mL. The median serum PSA level was 33.64 (0.44–860.9) ng/mL, the free PSA level was 4.56 (0.02–50) ng/mL, and the PSA density was 0.56 ng/mL (0.01–53.8). Reliability between ADC measurements was investigated using ICC. For intraobserver agreement, ICC was 0.816. The ICC for interobserver agreement was 0.879.

A negative correlation was found between ADC and SUVmax (*p* < 0.001, rho = −0.519). Also, there was a negative correlation between ADC and PSA (*p* < 0.001, rho = −0.372).

### 3.2. Evaluation by Gleason ISUP Grade

All parameters of ADC, SUVmax, SUVmax/ADC ratio, PSA, free PSA, and PSA density were compared separately according to each Gleason ISUP grade (1–2–3–4–5). No statistically significant difference was found as a result of ANOVA (*p* > 0.05).

Patients were divided into two groups: grades 1–2 (early) and grades 3–4–5 (advanced) according to ISUP grades. All parameters were analyzed according to the early- and advanced-grade groups.

ADC values between the early- and advanced-grade groups did not differ statistically significantly (*p* > 0.05).

**Figure 2:**
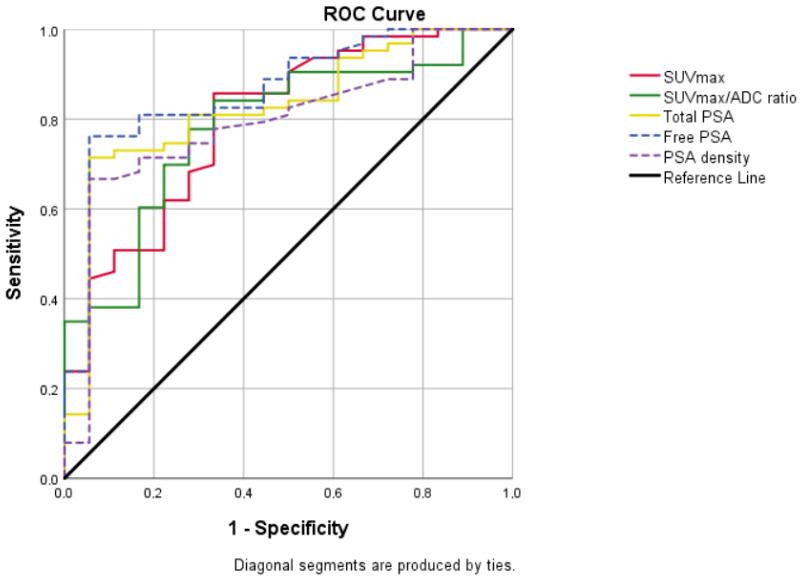
Graph showing all of the ROC curves which were made according to the early–advanced grade and were statistically significant. AUC was 0.797 for SUVmax (95% CI = 0.679–0.915; *p* < 0.001), 0.783 for SUVmax/ADC ratio (95% CI = 0.668–0.898; *p* < 0.001), 0.825 for total PSA (95% CI = 0.718–0.933; *p* < 0.001), 0.864 for free PSA (95% CI = 0.769–0.959; *p* < 0.001), 0.794 for PSA density (95% CI = 0.679–0.909; *p* < 0.001).

In terms of PSA, free PSA, SUVmax, SUVmax/ADC ratio, and PSA density values, there was a statistically significant difference between the early- and advanced-grade groups (*p* < 0.001) (Table 2). In the ROC analysis between early- and advanced grades, cut-off values were calculated as 8.09 for SUVmax, 16.29 for SUVmax/ADC ratio, 25.32 for PSA, 2.93 for free PSA, and 0.495 for PSA density. The area under the curve (AUC) was 0.797, 0.783, 0.825, 0.864, and 0.794, respectively (Figure 2).

ADC, apparent diffusion coefficient; AUC, area under curve; CI, confidence interval; PSA, prostate-specific antigen; SUVmax, standardized uptake value maximum; ROC, receiver operating characteristic.

### 3.3. Evaluation by Risk Group

In the risk classification recommended by the European Association of Urology (EAU) and other societies in the guideline, patients with prostate cancer are classified as low–intermediate–high-risk group in terms of both local recurrence and active surveillance according to PSA, ISUP grade, and clinical stage [4,21].

In this study, the number of intermediate-risk patients was 18, and the number of high-risk patients was 63. Therefore, the patients included in the study mainly consisted of intermediate-risk and high-risk patients.

**Figure 3:**
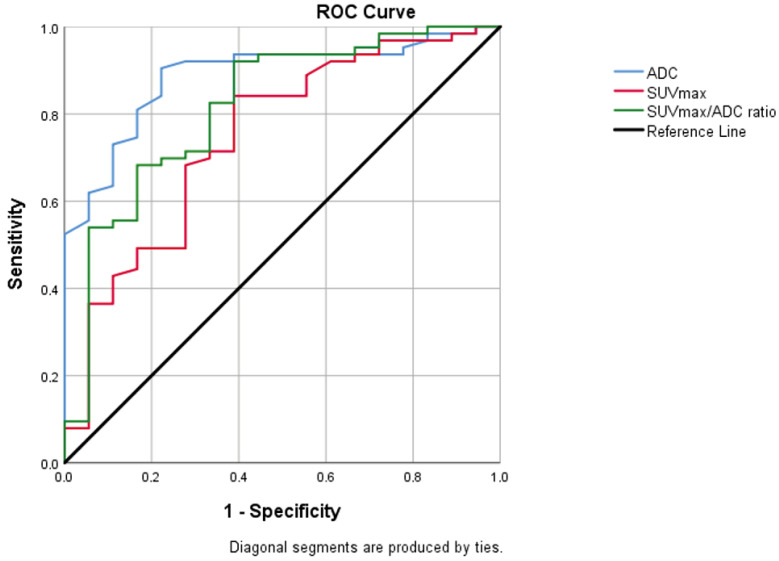
Graph of ROC analysis for ADC, SUVmax, SUVmax/ADC values between intermediate- and high-risk patient groups. AUC was 0.887 for ADC (95% CI = 0.810–0.965; *p* < 0.001), 0.74 for SUVmax (95% CI = 0.614–0.880; *p* = 0.001), 0.817 for SUVmax/ADC ratio (95% CI = 0.699–0.934; *p* < 0.001). ADC, apparent diffusion coefficient; AUC, area under curve; CI, confidence interval; SUVmax, standardized uptake value maximum; ROC, receiver operating characteristic.

In individuals with intermediate and high risk, there was a statistically significant difference between the values of ADC, SUVmax, and SUVmax/ADC ratio (*p* < 0.001) (Table 3). In the ROC analysis, the cut-off value was calculated as 0.52 for ADC, 9.73 for SUVmax, and 20.28 for SUVmax/ADC ratio (AUC = 0.887 [95% CI = 0.810–0.965], 0.747 [95% CI = 0.614–0.880], 0.817 [95% CI = 0.699–0.934], respectively) (Figure 3). Youden’s index was calculated as 0.643 for ADC, 0.405 for SUVmax, and 0.437 for SUVmax/ADC.

Logistic regression analysis revealed that both the model including individual ADC and SUVmax values (*p* = 0.006, AUC = 0.796 [95% CI = 0.675–0.917]) and the model using the SUVmax/ADC ratio as a sole variable (*p* = 0.002, AUC = 0.844 [95% CI = 0.749–0.949]) were significant predictors for the high-risk group.

### 3.4. Evaluation by CAPRA (Cancer of the Prostate Risk Assessment) Score

The relationship between the clinical risk assessed by the CAPRA score and the quantitative imaging parameters was investigated using Spearman’s rank correlation. A statistically significant, moderate negative correlation was found between the CAPRA score and ADC values (rho = −0.456, *p* < 0.001), indicating that higher clinical risk scores are associated with lower ADC values. Furthermore, the CAPRA score demonstrated a significant, moderate positive correlation with the SUVmax/ADC ratio (rho = 0.441, *p* < 0.001) and a significant positive correlation with SUVmax (rho = 0.359, *p* = 0.001).

### 3.5. Evaluation of Patients with Gleason Score of 7 and Intermediate-Risk Group

Patients with Gleason ISUP grade 2 (3 + 4) and grade 3 (4 + 3) with a Gleason score of 7 were also evaluated separately, and the parameters were compared.

There was a statistically significant difference in ADC, SUVmax, and SUVmax/ADC ratio values between grade 2 and 3 patients (*p* < 0.05).

Within the intermediate-risk group (*n* = 18), no statistically significant difference was observed between the favorable (*n* = 6) and unfavorable (*n* = 12) subgroups in terms of their ADC, SUVmax, and SUVmax/ADC ratio values (*p* = 0.29, *p* = 0.51, 0.28).

### 3.6. Evaluation by Perineural Invasion

There was no statistical significance in the comparison of the presence of perineural invasion, ADC, SUVmax, SUVmax/ADC ratio, PSA, free PSA, and PSA density values (*p* > 0.05).

At the same time, no statistically significant difference in perineural invasion was found in the comparison between the advanced-grade groups and high-risk patients (*p* > 0.05).

### 3.7. Evaluation by Bone Metastasis

In this study, a statistically significant correlation was found between the presence of bone metastases and PSA and free PSA levels (*p* = 0.003, *p* = 0.001, respectively). Accordingly, the cut-off value was calculated as 27.97 for PSA and 3.85 for free PSA (AUC = 0.693, AUC = 0.710, respectively).

A total of 31 patients had bone metastases, of whom 24 were classified as oligometastatic and 7 were classified as polymetastatic (>3 metastases). A comparison between the oligometastatic and polymetastatic groups, which were stratified by the number of bone metastases, revealed no statistically significant differences in the values of ADC, SUVmax, and SUVmax/ADC ratio (*p* = 0.29, *p* = 0.28, and *p* = 0.63, respectively).

### 3.8. Evaluation by Lymph Node Metastasis

In this study, a statistically significant correlation was found between the presence of lymph node metastases and SUVmax, SUVmax/ADC ratio, PSA, and free PSA values (*p* = 0.019, *p* = 0.01, *p* = 0.034, *p* = 0.043, respectively). Accordingly, the cut-off value was calculated as 11.63 for SUVmax, 23.6 for SUVmax/ADC ratio, 34.26 for PSA, and 4.78 for free PSA (AUC = 0.655, AUC = 0.670, AUC = 0.640, AUC = 0.633, respectively).

### 3.9. Survival Analysis

The median follow-up period for the entire cohort of 81 patients was 62 months (range: 3–128 months). During this period, 35 patients (43.2%) died from any cause. Median overall survival (OS) was 57 months.

**Figure 4:**
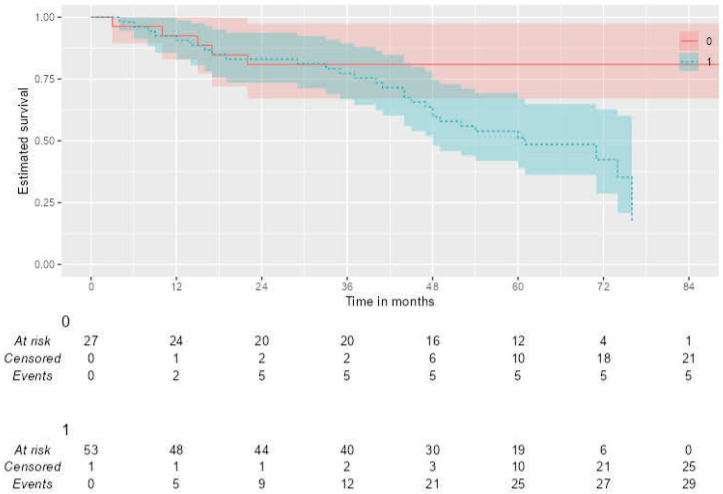
Kaplan–Meier curves for Overall Survival (OS) stratified by the ADC value. Patients were dichotomized based on a pre-defined ADC cut-off of 0.52 × 10^−3^ mm^2^/s. The analysis shows that (1) low ADC group (*n* = 50) had significantly poorer overall survival compared to (0) high ADC group (*n* = 31) (Log-rank test, *p* = 0.013).

Kaplan–Meier analysis was performed to assess the impact of quantitative imaging parameters on overall survival. Patients with low ADC values (<0.52) had a significantly shorter median overall survival compared to patients with high ADC values (106 vs. 61 months; Log-rank *p* = 0.013) (Figure 4). However, no significant difference in OS was observed based on the SUVmax cut-off value (>9.73), high SUVmax/ADC ratio (>20.28) (Log-rank *p* =0.974, *p* = 0.470).

In the univariate Cox regression analysis, a low ADC value (HR: 3.16; 95% CI: 1.22–8.20; *p* = 0.018), was significantly associated with worse overall survival.

## 4. Discussion

It has been demonstrated that Ga-68 PSMA PET/CT is more sensitive and effective than mpMRI in identifying tumor location and index lesions [22,23,24,25]. Therefore, in this study, the use of Ga-68 PSMA PET/CT was deemed appropriate for index lesion detection.

Diffusion-weighted sequences are essential for imaging prostate cancer. In ADC maps, tumor foci are seen at a lower signal than in benign prostate tissue [11]. It has been demonstrated that tumor cellularity affects ADC values and that ADC values are lower in tumors with high cellularity [26].

The mean ADC value measured from malignant tumor foci was calculated as 1.30 ± 0.30 × 10^−3^ mm^2^/s in Desouza et al. [27], 1.45 ± 0.26 in Zelhof et al. [26], and 0.72 ± 0.27 in Chinnappan et al. [28].

In this study, the mean of the ADC values was measured as 0.49 ± 0.11 × 10^−3^ mm^2^/s, which was lower than the data in the literature. In this study, for ADC measurement, the index/dominant lesion was determined by PET/CT and measurements were made from this lesion. The lower measurements compared to the literature were attributed to the easier differentiation of the borders and sizes of index/dominant lesions in PET/CT.

In this study, there was a significant correlation between ADC and Gleason grade (*p* < 0.001, r = −0.477), the same as in [29]. This demonstrates a correlation between the ADC value and the prostate cancer’s histological grade.

According to Sökmen et al. [30], the high-risk group’s ADC values were found to be statistically considerably lower than those of the other groups (*p* < 0.001). In studies investigating the SUVmax cut-off value for predicting high-risk prostate cancer, Nuo et al. [31] reported a threshold of 12.9, while Ulas Babacan et al. [32] determined a value of 8.75. In present study, we established this cut-off value at 9.73.

In this study, a statistically significant difference was found between the two groups in terms of measured ADC, SUVmax, and SUVmax/ADC values (*p* < 0.001). For intermediate–high-risk discrimination, the cut-off values were found to be 0.52 for ADC, 9.73 for SUVmax, and 20.28 for SUVmax/ADC ratio (AUC = 0.887, AUC = 0.747, and AUC = 0.817, respectively). Thus, it is thought that ADC and SUVmax measurements can predict a high risk of the disease.

Studies by Zhang et al. [33] and Wang et al. [34] both concluded that AUC for the SUVmax/ADC ratio was higher than for its individual components in differentiating malignant from benign disease. Zhang et al. [33] proposed an optimal cut-off value of 15 to identify high-risk patients. Furthermore, this metric demonstrates a significant association with metastatic potential. A higher SUVmax/ADC ratio in the primary tumor has been linked to the presence of nodal and distant metastases, with one study suggesting a value greater than 27 as a predictor for metastatic disease.

While the exact cut-off values for diagnosing prostate cancer vary slightly between cohorts—ranging from 7 and 7.43 in the studies by Zhang et al. [33] and Chinnappan et al. [28], to 12.35 in the study by Wang et al. [34]—the overarching conclusion is that a higher ratio is strongly indicative of malignancy.

In the logistic regression model performed in this study, the SUVmax/ADC ratio, when considered as a standalone variable, was found to be a more powerful predictor for identifying high-risk patients compared to the model where ADC and SUVmax were evaluated separately (AUC = 0.844 vs. 0.796, respectively). However, we acknowledge that this analysis does not definitively prove whether the ratio provides an independent and additional prognostic value over ADC and SUVmax. To clarify the incremental value of this ratio, future studies with larger patient cohorts are needed, incorporating multivariate analyses that include all three variables.

The findings of the present study demonstrate a significant and moderate correlation between the CAPRA score, a validated clinical risk assessment tool, and quantitative imaging biomarkers, which provides substantial validation for ADC, SUVmax, and SUVmax/ADC ratio as non-invasive indicators of biological aggressiveness in prostate cancer. It is noteworthy that these correlations, while significant, are moderate rather than perfect. This suggests that while imaging markers and clinical scores measure related aspects of tumor biology, they are not merely redundant proxies for one another. Instead, imaging appears to offer unique insights not fully captured by standard pathological and clinical data. This foundational finding was essential for subsequent analysis, where we demonstrated that integrating imaging data with the CAPRA score significantly enhanced predictive accuracy, highlighting that the most powerful prognostic assessment may lie in the synergistic combination of these tools.

Chen et al. [35], in their study, determined that SUVmax values measured in Ga-68 PSMA PET/CT showed a positive correlation with Gleason grade, PSA levels, and tumor volume (*p* < 0.01). In their study, the cut-off SUVmax value for early–advanced prostate cancer was calculated as 8.4. In this study, this value was calculated as 8.09, similar to Chen et al.

In another study that measured SUVmax, values were grouped as ISUP grades 1–2 and 3–4–5 [36], and SUVmax and grade had a statistically significant difference. According to a cut-off SUVmax value of 18.78, the Youden index was 0.57 (*p* = 0.008, AUC = 0.84). In another study conducted according to the same grouping [37] with a cut-off SUVmax value of 11.4, the Youden index was 0.28 (AUC = 0.65). In this study, the cut-off SUVmax value was found to be 8.09 and the Youden index was 0.52 (*p* < 0.001, AUC = 0.797).

The results are consistent with the literature and show that the SUVmax value can predict advanced disease (Table 4).

In evaluating ISUP grade 2 (3 + 4) and grade 3 (4 + 3) patient groups with a Gleason score of 7, ADC, SUVmax, and the SUVmax/ADC ratio were significantly different between these two groups (*p* < 0.05).

Kim et al. [38] found a statistically significant difference with the ADC value for the presence of extracapsular extension (*p* < 0.001, AUC = 0.771). The prognosis and extraprostatic prostate cancer spread are closely correlated with the existence of perineural invasion [1]. The occurrence of perineural invasion was shown to be associated with elevated PSA levels (*p* = 0.02) [39]. However, perineural invasion was not found to be significantly associated with other parameters, including ISUP grade. Another research study on perineural invasion [40] discovered that the existence of perineural invasion was strongly related to mortality; however, it was not found to be associated with age, ISUP grade, or tumor volume (*p* = 0.17). There was no statistically significant relationship between the occurrence of perineural invasion and variables such as ADC, SUVmax, SUVmax/ADC ratio, prostate volume, age, PSA, free PSA, and PSA density in this research (*p* > 0.05).

A statistically significant relationship was discovered between the occurrence of bone metastases and PSA value in a study of prostate cancer cases with bone metastases [41]. In this study, PSA and free PSA values were statistically highly associated with bone metastases (*p* < 0.05). It is understood that PSA is still a strong prognostic marker for distant metastasis.

It is known in the literature that SUVmax values on PSMA PET/CT are used in the assessment of bone and lymph node metastases [42,43]. In comparison with these studies, this study reveals the importance of the SUVmax value in intraprostatic disease and demonstrates its low sensitivity in detecting lymph node metastasis [44,45]. In light of these data, it is emphasized that larger patient cohorts are necessary, particularly for the evaluation of lymph node metastasis.

Contrary to our hypothesis, we did not find a statistically significant association between the primary tumor’s quantitative imaging parameters (ADC, SUVmax, and SUVmax/ADC ratio) and the burden of bone disease (oligometastatic vs. polymetastatic). This lack of a clear relationship is likely attributable to the limited statistical power of this subgroup analysis given the small sample size. Therefore, while our biomarkers may be indicative of metastatic potential in general, larger prospective studies are warranted to determine whether imaging features of the primary tumor can specifically predict the extent of future metastatic dissemination.

In recent years, studies have investigated the cut-off values of ADC for benign–malignant differentiation in prostate lesions [46,47,48,49]. However, there are relatively few studies investigating the effect of low ADC values on survival. One study using whole-body DWI found that a low ADC value in prostate cancer was associated with shorter survival times [50]. The present study demonstrates that a low ADC value serves as significant independent predictors of worse overall survival in patients with high-risk prostate cancer. The impact of low ADC on survival in this study (HR: 3.16) is comparable to previous findings, such as the study by Fujiwara et al. [50] which reported a hazard ratio of 4.0.

It is well-established that the Gleason score in prostate cancer can be susceptible to sampling errors inherent in biopsy, potentially leading to undergrading of the tumor. Quantitative parameters such as ADC and SUVmax, which non-invasively assess the entire tumor volume, may therefore serve as valuable adjuncts to mitigate this risk. For instance, in a patient with a ISUP Grade Group 2, discordant imaging findings, such as a very low ADC value and a very high SUVmax, could indicate the presence of a more aggressive tumor than suggested by pathology, potentially harboring an unsampled component of a higher Gleason pattern. Such information could consequently influence the therapeutic strategy. Furthermore, these biomarkers may hold promise for post-treatment surveillance, for example, in the setting of biochemical recurrence, where they could aid in guiding salvage therapy decisions.

Firstly, the retrospective and single-center design of this study may have introduced potential selection bias and limited the generalizability of the results to other centers. Second, the sample size of 81 patients is relatively small, which may have reduced the statistical power, particularly in subgroup analyses.

One of the most significant limitations of this study is that approximately half of the patient cohort sample consists of patients in the high-risk group and those with aggressive disease. This is due to the strict selection criteria for including low-risk patients under the social security coverage for Ga-68 PSMA PET/CT scans in the country. Consequently, the cut-off values and results we obtained are particularly valid for moderate- and high-risk patient populations and should not be generalized to low-risk or screening populations.

The absence of a PET/MRI device in the clinic is another limitation. Today, these devices are available in very few centers due to cost and usage areas. It might be more beneficial to conduct future studies by correlating PET/MRI, which has a higher sensitivity than PET/CT.

A limitation of the survival analysis is the median follow-up period of 62 months, which may be insufficient to capture late events. This analysis was based on overall survival, and cause-of-death data was not uniformly available. Additionally, the data set does not include the treatment method applied for prostate cancer (hormonal, focal, radiotherapy, surgery, etc.). Therefore, we cannot definitively attribute all mortality events to prostate cancer progression, as deaths from comorbidities could be a confounding factor.

Given the negative correlation we found between ADC and SUVmax, a potential collinearity exists between these two variables and the derived SUVmax/ADC ratio. Although this did not directly affect the analysis as we did not construct a multivariate model, the issue of multicollinearity must be addressed in the future. When developing prognostic models that include all three parameters, multicollinearity should be formally evaluated using methods such as the variance inflation factor (VIF).

## 5. Conclusions

In conclusion, it was determined that quantitative data acquired from Ga-68 PSMA PET/CT and mpMRI examinations for patient staging can be used to predict outcomes. When correlated with Ga-68 PSMA PET/CT, it becomes simpler to isolate and identify the index lesion on mpMRI. Quantitative ADC, SUVmax, and SUVmax/ADC measurements are promising for identifying high-risk cases in the future.

## Figures and Tables

**Table 1:** Patient demographics.

	*n* = 81	%
**Gleason ISUP grade**		
Grade 1	6	7.4
Grade 2	12	14.8
Grade 3	12	14.8
Grade 4	11	13.5
Grade 5	40	49.3
**Perineural invasion**		
Present	51	63
Absent	30	37
**Bone metastasis**		
Present	31	38.2
Absent	50	61.8
**Lymph node metastasis**		
Present	32	60.4
Absent	49	39.6
**Risk group**		
Intermediate risk	18	22.2
High risk	63	77.8

ISUP, International Society of Urogenital Pathologists.

**Table 2:** Parameters evaluated according to early–advanced-grade groups.

Parameter	*p* Value	Cliff Delta (Effect Size)	95% CI	Cut-Off	AUC	Sensitivity	Specificity
ADC	0.18						
SUVmax	0.0001	−0.594	−0.812, −0.335	8.09	0.797	85%	66%
SUVmax/ADC	0.0002	−0.566	−0.816, −0.335	16.29	0.783	84%	66%
PSA	2.98 × 10^−5^	−0.649	−0.79, −0.31	25.32	0.825	71%	94%
Free PSA	2.65 × 10^−6^	−0.728	−0.892, −0.514	2.93	0.864	76%	94%
PSA density	0.0001	−0.587	−0.79, −0.322	0.49	0.794	66%	94%

ADC, apparent diffusion coefficient; AUC, area under curve; CI, confidence interval; PSA, prostate-specific antigen; SUV, standardized uptake value.

**Table 3:** Parameters evaluated according to intermediate–high-risk discrimination.

Parameter	*p* Value	Cliff Delta (Effect Size)	95% CI	Cut-Off	AUC	Sensitivity	Specificity
ADC	5.25 × 10^−8^	0.536	0.810, 0.965	0.52	0.887	81%	83%
SUVmax	0.0006	−0.442	−0.614, −0.880	9.73	0.747	68%	72%
SUVmax/ADC	0.0002	−0.498	−0.699, −0.934	20.28	0.817	71%	72%

ADC, apparent diffusion coefficient; AUC, area under curve; CI, confidence interval; SUV, standardized uptake value.

**Table 4:** Studies showing the relationship between cut-off SUVmax values measured in separate groups as Gleason ISUP grade 1–2 (early) and 3–4–5 (advanced). (ISUP, International Society of Urogenital Pathologists; SUV, standardized uptake value).

	Cut-Off SUVmax	AUC	Sensitivity	Specificity	Youden Index
Jochumsen et al. [36]	18.78	0.84	76%	81%	0.57
Klingenberg et al. [37]	11.4	0.65	51.2%	77.4%	0.28
This study	8.09	0.79	85.7%	66.6%	0.52

AUC, area under curve; SUV, standardized uptake value.

## Data Availability

Upon reasonable request, the authors will make the datasets analyzed in the current work available.
